# Supplementary material for: Protective Effect of Zeaxanthin from Lycium barbarum L. on Ultraviolet B-Induced Skin Photodamage in Mice Through Nrf2-Related Pathway
Source: Antioxidants (Basel). 2025 May 25;14(6):632. doi: 10.3390/antiox14060632 (PMC12189396; doi:10.3390/antiox14060632)
Supplement: Supplementary file 1 [file antioxidants-14-00632-s001.zip › antioxidants-3641113-supplementary.pdf]

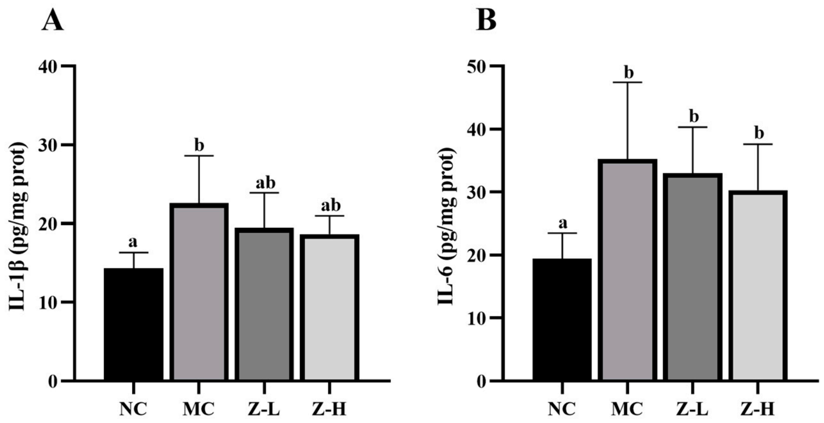

Figure S1. The content of inflammatory factors in skin tissues of mice. (**A**) IL-1β; (**B**) IL-6. Different letters indicated significant differences at $p < 0.05$. Data were expressed as mean ± SEM (n = 5–6).
